# The Hospital

**Published:** 1896-04-11

**Authors:** 


### The Hospital.

AN

### Institutional Journal of the Medical Sciences and Hospital Administration.

**Weekly 2d.** **Monthly 6d.**

**WITH AN EXTRA NURSING SUPPLEMENT OF 20 PAGES.**

Published Weekly. Price **2d.** Publishing and Editorial Offices: 428, Strand, London, W.C.

The success which has attended the publication of "THE HOSPITAL" newspaper, and its unique circulation amongst the staffs of the hospitals and medical institutions, not only in this country, but in Greater Britain, India, and the United States of America, have encouraged the Editors to continue to use every effort to render it the most generally useful medical organ to the great body of medical practitioners.

Dr. Pye Smith, in the Harveian Oration last year, pointed out that "there is all the difference between learning the present conclusions as they stand in the last edition of a text-book or compendium, and tracing the steps by which our present knowledge has been reached." The conductors of "THE HOSPITAL," in the new departure which they are making in medical journalism, have determined to reduce this exhortation to practice by securing the co-operation of eminent members of the medical profession who are also able writers and close observers, who will describe the progress of the science of medicine, and trace the steps by which our present knowledge has been reached.

With this view arrangements have been made for series of articles (and which are now appearing) on

"MODERN THERAPEUTICS," by Sir Benjamin Ward Richardson, M.D., F.R.S.;

On "MODERN PROGRESS IN OPHTHALMIC MEDICINE AND SURGERY," by Mr. R. Brudenell Carter, F.R.C.S., Consulting Ophthalmic Surgeon to St. George's Hospital;

On "SOME ASPECTS OF MODERN PSYCHOLOGY," by Dr. Clouston, F.R.C.P., Edin., Lecturer on Mental Diseases to the University of Edinburgh; and

On "MODERN BIOLOGY," by Mr. J. Bretland Farmer, M.A., F.L.S., Assistant Professor of Biology, Royal College of Science, South Kensington; in addition to articles on other subjects, which will be treated by physicians, surgeons, and scientists of eminence.

And for many other articles of special interest, all written by experts.

One of the new departments in "THE HOSPITAL" is entitled "MEDICAL PROGRESS," and provides the profession with a concise, authoritative, and readable epitome of the most recent advances in medical science.

In addition to original contributions upon treatment, practice, and therapeutics, each number contains a comprehensive record of progress in one or more branches of medicine, surgery, laryngology, obstetrics, gynæcology, ophthalmology, and other special departments, so as to secure the whole field of medicine being covered, in the widest and fullest meaning of the term.

These articles are not prepared from a critical or partizan point of view, but are made to embrace the chief points of every paper and contribution of importance, so that each worker, in his own particular branch will here find a complete record of all the work done by others, wherever trained practitioners are trying to solve the problem of disease.

It may further be added that the department of "MEDICAL PROGRESS" will be strengthened by ample and precise references to the original papers and sources of information.

No expense has been, or will be, spared to make "MEDICAL PROGRESS AND HOSPITAL CLINICS" exhaustive and complete.

The practical value of this new departure in medical journalism must be patent to every

*For Subscription Form see next page.*

practitioner, and it will fill a place unoccupied by any other publication hitherto produced in the English language.

The true interests of the majority of medical practitioners are much concerned with the wise ADMINISTRATION OF HOSPITALS and medical institutions. "THE HOSPITAL" deals with the various questions involved in all their bearings, and so helps to form and lead medical and public opinion on these subjects.

The WORKSHOP SECTION contains plans of all the most recent improvements and developments in hospital construction, including drainage and sanitation, matters which seriously affect the welfare of the patients, and are becoming every day of more importance and interest to the medical profession. The reports on hospital administration by "THE HOSPITAL'S" special commissioner, containing an account of the present condition and work of the chief British and foreign hospitals, have already excited a wide interest, and have proved not only popular but instructive to all who are interested in the subject.

"THE HOSPITAL" has already acquired an influence and circulation which has given it a foremost place in medical journalism, and this fact, coupled with the representations which have been made to the Publishers by influential and representative members of the medical profession, point to the conclusion that the many new developments recently determined upon, and especially the department devoted to "MEDICAL PROGRESS AND HOSPITAL CLINICS," will command a warm and extended welcome, and so secure that "THE HOSPITAL," which already commands the largest *bonâ fide* sale of any English medical journal, will speedily possess the greatest circulation also.

